# Resveratrol, novel application by preconditioning to attenuate myocardial ischemia/reperfusion injury in mice through regulate AMPK pathway and autophagy level

**DOI:** 10.1111/jcmm.17431

**Published:** 2022-07-05

**Authors:** Haiyan Li, Fuchun Zheng, Yanmei Zhang, Jiajia Sun, Fenfei Gao, Ganggang Shi

**Affiliations:** ^1^ Department of Pharmacology Shantou University Medical College Shantou China; ^2^ Reproductive Center of the First Affiliated Hospital of Shantou University Medical College Shantou China

**Keywords:** AMPK, autophagy, FOXO1, myocardial ischemia/reperfusion injury, resveratrol, SIRT1

## Abstract

Myocardial ischemia/reperfusion injury (MI/RI) is the main cause of deaths in the worldwide, leading to severe cardiac dysfunction. Resveratrol (RSV) is a polyphenol plant‐derived compound. Our study aimed to elucidate the underlying molecular mechanism of preconditioning RSV in protecting against MI/RI. Mice were ligated and re‐perfused by the left anterior descending branch with or without RSV (30 mg/kg·ip) for 7 days. Firstly, we found that RSV pretreatment significantly alleviated myocardial infarct size, improved cardiac function and decreased oxidative stress. Furthermore, RSV activated p‐AMPK and SIRT1, ameliorated inflammation including the level of TNF‐α and IL‐1β, and promoting autophagy level. Moreover, neonatal rat ventricular myocytes (NRVMs) and H9c2 cells with knockdown the expression of AMPK, SIRT1 or FOXO1 were used to uncover the underlying molecular mechanism for the cardio‐protection of RSV. In NRVMs, RSV increased cellular viability, decreased LDH release and reduced oxidative stress. Importantly, Compound C(CpC) and EX527 reversed the effect of RSV against MI/RI in vivo and in vitro and counteracted the autophagy level induced by RSV. Together, our study indicated that RSV could alleviate oxidative stress in cardiomyocytes through activating AMPK/SIRT1‐FOXO1 signallingpathway and enhanced autophagy level, thus presenting high potential protection on MI/RI.

## 1 INTRODUCTION

Myocardial ischemia and hypoxia usually caused by coronary artery stenosis and occlusion. Emergency intervention surgery can quickly remove sick blood from the blood vessel, which restores the blood oxygen supply of the ischemic myocardium is the main treatment methods of acute myocardial infarction (AMI) in the treatment window.^1^, ^2^ However, the blood supply was restored, reperfusion of blood flow in the ischemic area will also cause further damage to cardiomyocytes called myocardial ischemia–reperfusion injury. MI/RI is involved in intracellular calcium overload, mitochondrial dysfunction, oxidative stress, inflammation reaction and can develop into irreversible cell death through apoptosis.^3^, ^4^, ^5^

RSV is a kind of polyphenol antioxidant, mainly found in grapes, berries, peanuts and other fruits. Since RSV was discovered, it has been valued by many scientists. With the in‐depth study of RSV, research's shown that RSV has a wide range of pharmacological actions. Such as, anti‐oxidation,^6^ anti‐cardiovascular disease,^7^ liver protection,^8^ anti‐cancer,^9^ antibacterial,^10^ anti‐inflammatory^11^ and anti‐aging.^12^

Recent studies have pointed out that silent information regulator of transcription 1 (SIRT1) acts as pre‐sensor of body energy metabolism, targeting histones, co‐regulatory molecules, and metabolic enzymes to regulate gene expression and metabolic activity, thus reflecting the energy state of the cells.^13^ SIRT1 is located in the cytoplasm and nucleus and has various of interacting proteins, including AMP‐activated protein kinase (AMPK), forkhead transcription factor 1 (FOXO1) and other sensory proteins related to energy metabolism.^14^, ^15^ Their interaction forms an energy sensor network affecting body energy metabolism, such as glycolipid metabolism, mitochondrial quality control, insulin secretion and metabolic function, etc.^16^

AMPK is a kinase that sense cell energy levels and plays an important role in cell metabolism.^17^ AMPK and SIRT1 are both cellular energy‐sensing proteins, while their action mechanism are different. When cell energy is deficient, activation of AMPK restores energy balance by stimulating the catabolic processes that produce ATP and downregulating the assimilation processes that consume ATP. SIRT1 prolongs the body's lifespan indirectly regulates energy restriction, but it also acts on many cells biological processes such as energy metabolism and mitochondria, which consistent with AMPK.^18^ Previous research shows that both AMPK and SIRT1 have been activated under calorie restriction and body stress.^19^ When AMPK activated, it can upregulate the expression of NAMPT and decrease the content of nicotinamide to increase the content of NAD+ in the cell and enhance the activity of SIRT1 stresses such as calorie restriction and exercise, and they can be mutually regulated. At the same time, SIRT1 can increase the activity of AMPK by deacetylating liver kinase B1 (LKB1), which is an upstream kinase of AMPK.^20^

Based on these considerations, we innovatively observed the effect of RSV on MI/RI and investigated the underlying mechanism. We found RSV increased myocardium energy metabolism and reduce excessive ROS production in cardiomyocytes by activating AMPK/SIRT1 signalling pathway, and effectively enhanced autophagy level. Moreover, Compound C(CpC) and EX527 reversed the effect of RSV against MI/RI in vivo and in vitro and counteracted the autophagy level induced by RSV. Our research might present a new strategy to decrease the damage caused by ischemic heart disease.

## 2 MATERIALS AND METHODS

### 2.1 Antibodies and reagents

RSV, 2,3,5‐triphenyltetrazolium chloride (TTC) and dimethyl sulphoxide (DMSO) were obtained from Sigma Aldrich. The haematoxylin and eosin (H&E) staining kit (Cat#SBJ‐1247) and RIPA lysis buffer (#P0013B) were obtained from Beyotime Biotechnology. The primary antibodies used are as follows: p‐AMPK (#4188), AMPK (#5831), SIRT1(#9475), p‐FOXO1(#9461) FOXO1(#2880), LC3B (#2775), Beclin‐1 (#3495), Atg12(#4180), P62(#5114) were obtained from Cell Signaling Technology. β‐actin and β‐Tubulin from ZSGB‐BIO. EX527(HY‐15452) and Compound C (HY‐13418A) were obtained from MCE. HRP‐conjugated goat anti‐mouse/rabbit secondary antibodies came from ZSGB‐BIO. Prime Scripttr RT reagent Kit with gDNA Eraser (# AK3801) and SYBR Premix Ex TaqTMII (# AKA303) were from Takara. TNF‐α ELISA Kit (EK0527) and IL‐1β ELISA Kit (# EK0394) and were obtained from Wuhan Boster Biological Technology Ltd. LDH, SOD and MDA kits were purchased from Nanjing Jiancheng. Lipofectamine^TM^2000 (Lip2000) was obtained from Invitrogen Life Technologies. Bromodeoxyuridine (Sigma). DAPI solution (#C1005, Beyotime).

### 2.2 Animals

Adult male C57BL/6J mice (20–25 g) were obtained from Beijing Vital River Laboratory Animal Technology and were housed at a well‐controlled environment (23°C ± 2°C; ventilation, 12 h light and 12 h dark) with free access to food and water. All experiments were complied with the NIH Guide for “The Care and Use of Laboratory Animals” and were approved by the animal care committee of the Shantou university medical college. After 7 days of acclimatization, mice were randomly divided into Sham, I/R + Vehicle and I/R + medication groups (*n* = 5 to 6) mice for each experimental group. RSV was dissolved in 1% dimethyl sulfoxide/sterile water for injection. Prior to I/R surgery, the I/R + RSV (30 mg/kg) group, I/R + RSV (30 mg/kg) + CpC(20 mg/kg) and I/R + RSV (30 mg/kg) + EX527(5 mg/kg) groups received an intraperitoneal injection RSV 30 mg/kg once daily for 7 days and once again prior to operation. CpC 20 mg/kg and EX527 5 mg/kg were intraperitoneally administrated 20 min before reperfusion. The vehicle group received the same volume of DMSO without RSV. Medication doses were chosen according to previous studies.^21^, ^22^, ^23^, ^24^

### 2.3 Myocardial I/R injury model

We performed Murine MI/RI model as previously described.^25^ Briefly, mice were anaesthetised with pentobarbital (50 mg/kg, i.p.) and ligated the mouse left anterior descending artery (LAD). The electrocardiogram showed ST‐segment elevation and the myocardial tissue supplied by ischemic vessels changed from bright red to pale in mice indicated mice were in a state of ischemia. After 30 min of LAD ligation, release the ligature and allow the mouse to reperfusion. The echocardiography was used to measure mouse cardiac function, and myocardial tissues and serum were harvested and stored at −80°C for testing of other indicators.

### 2.4 Myocardial infarct size and HE staininganalysis

Infarct size after MI/RI was examinate by Evens‐TTC staining. In detail, after 24 h reperfusion, mice were anaesthetised, the LAD artery was re‐occluded at the previous ligation, 1000 μl of Evans blue (1%) were injected into the left ventricle cavity and removed the mouse hearts rapidly by resection, the heart was cleaned and squeezed, dipped in dried blood, rinsed with saline at 4°C, dipped in dry hearts were resected, and then 10 min snap‐frozen at −80°C. Subsequently, the myocardium was uniformly cut into 4 to 5 pieces below the ligation site and then immersed in 1% TTC incubation (37°C, 15 min). Photographs were taken with scanner (Panasonic) and quantified by Image J software (Media Cybernetics).

For hematoxylin‐eosin (H&E) staining analysis, pre‐chilled PBS buffer was used to wash away blood from the myocardium, then 4% paraformaldehyde fixation (4°C, 24 h), then dehydrated in graded ethanol, cleared with xylene and embedded in paraffin. Samples taken 2 mm below the ligature were then serially sectioned at 4 μm thickness and stained with H&E kit. Photograph the slides under a microscope (Zeiss Microsystems).

### 2.5 Echocardiographic analysis

After 24 h reperfusion, Mice were anaesthetised by breathing in 1%–2% isoflurane, allowing for noninvasive examination and put on a heating plate to maintain body temperature. Echocardiography equipment system (Visual Sonic Vevo 2100) used to assesse cardiac function by using a 15‐MHz linear transducer. Left ventricular end‐diastole diameter (LVEDd) and left ventricular end‐systolic diameter (LVESd) were measured, respectively. M‐mode echocardiograms were saved and used to detect cardiac function of mice. Fractional shortening (LVFS) and ejection fraction (LVEF) and were calculated using vevo LAB 3.1.0.

### 2.6 Cell culture and treatment

Using a modification of a previously described protocol,^26^ NRVMs were isolated from whole hearts of neonatal 1–3 day old Sprague–Dawley rats. Briefly, hearts were minced and digested with 0.25% trypsin for 12 h, then terminate digestion with Dulbecco's modified Eagle's medium (DMEM) containing 10% foetal bovine serum (FBS), washed 3 times with PBS, and then digest with Type II collagenase (gibco, Thermo Fischer Scientific) at 37°C. The digested cell suspension was pre‐plated to clear fibroblasts. NRVMs were cultured in DMEM complete medium supplemented with 1% antibiotic‐antimycotic mix and 100 μM bromodeoxyuridine (Sigma). then after 3–4 days cells were subjected to H/R procedure as flowing group.

NRVMs were randomly divided into three groups: (1) Control group, NRVMs were incubated with 95% mixed air and 5% CO_2_ in 0.1% FBS DMEM during experimental period. (2) H/R + Vehicle group, NRVMs were exposed to hypoxia buffer pH 6.2: 0.9 mM CaCl_2_, 4 mM HEPES and 20 mM Na lactate 137 mM NaCl,12 mM KCl, 0.49 mM MgCl_2_ 6H_2_O, by transferring the culture plates to hypoxic/anaerobic workstation with 94% N_2_, 5% CO_2_ and 1% O_2_ for 2 h, and then switched to 0.1% FBS DMEM and condition with 5%CO_2_ and 95% mixed air for reoxygenation for 2 h. (3) H/R + RSV group, NRVMs were pretreated with RSV at a concentration of 30 μM for 24 h before hypoxia and then underwent H/R protocol same as the H/R group. (4) To explore the effect of AMPK, SIRT1 in alleviating H/R damage, NRVMs were treated with 20 μM AMPK inhibitor (Compound C), or 10 μM SIRT1 inhibitor (EX‐527) at the beginning of reoxygenation, respectively. The dose was chosen according to previous studies.^27^, ^28^

H9c2 cells were seeded at a density of 1 × 10^4^/cm^2^ and cultured at DMEM containing 10% FBS with antibiotics in a cell incubator (humidified, 95% mixed air, 5% CO_2_, 37°C). For RNA interference, the siRNA sequences used to silence rat AMPKα (5′‐GCAUAU GCUGCAGGUAGAUdTdT‐3′), SIRT1 (5′‐UGAAGUGCCUCAGAUAUUA‐3′) and FOXO1 (5′‐CCAGGCACCUCAUAACAAA‐3′) or control scramble siRNA were transfected with Lip 2000 for 6 h in Opti‐MEM medium. After the transfection procedure, cells were transferred to full‐growth medium for another 24 h and processed as group divided, mRNA level detect the transfection efficiency and 48 h protein analysed for further studies.

### 2.7 Serum LDH, cTnI, CK‐MB and inflammation indicates analysis

After reperfusion 4 h, mice blood samples were harvested, placed in sterile EP tubes with heparin, and centrifuged at 3000 *g* for 15 min at 4°C to harvest serum. And then serum was separated to detect myocardium damage indicators, such as lactic dehydrogenase (LDH), creatine kinase (CK) and creatine kinase‐MB (CK‐MB). The automatic chemistry analyser (Toshiba Medical Systems Corporation) was used to measure these indicators.

Cardiomyocytes seeded at a density of 5 × 10^4^ cells/cm^2^ in a 6‐well plate. According to experimental requirements, after reoxygenation 2 h, the culture medium samples were collected. LDH release was detected by a lactate dehydrogenase assay kit (Nanjing Jiancheng) according to the manufacturer's instructions. Meanwhile, cell supernatant was also used to determine the concentrations of TNF‐α and IL‐1β detected by ELISA kit.

Following a previously described protocol, detect the concentration of TNF‐α and IL‐1β in myocardium tissue.^29^ Brifely, after the mice reperfusion for 4 h, the protein was collected the LV myocardial tissue below the ligature, including the non‐infarcted area and the scarred area. 20 mg myocardial tissue sample was homogenized in 200 μl of 1 × PBS (pH = 7.4) and then stored at −80°C overnight. After two freeze–thaw cycles to dissociate cell membranes, centrifuge the homogenate at 9660 *g* for 15 min. Samples were analysed according to the manufacturer's procedures.

### 2.8 RT‐PCR analysis

Neonatal rat ventricular myocytes total RNA was separated and extracted by Trizol (TaKaRa Biotechnology). RNA concentration quantities by nanodrop2000 (Thermo Fisher Scientific). PrimerScript® RT reagent Kits with gDNA Eraser Kits (TaKaRa Biotechnology) were used to reverse 1 μg total RNA transcribed into cDNA. RT‐PCR experiment was performed by SYBR® Premix ExTaq™ II kits (TaKaRa Biotechnology) on an ABI LIFE QuantStudio 12 K detection system (Applied Biosystems). Total reaction volume was 10 μl contained 1 μl cDNA in a template. The primers used are listed in Table 1 and the relative mRNA expression in each group was detected by using the comparative Ct (2^−ΔΔCT^) method in reference to GAPDH. A melting curve of each amplicon was determined to verify its specificity.

**TABLE 1:** Primers used for real‐time PCR in this study

Genes	Forward primer sequence	Reverse primer sequence
TNF‐α	5′‐gtcgtagcaaaccaccaagc‐3′	5′‐tgtgggtgaggagcacatag‐3′
IL‐1β	5′‐gcaatggtcgggacatagtt‐3′	5′‐agacctgacttggcagaga‐3′
AMPK	5′‐tttgcctagaatccccgcga‐3′	5′‐taaggagcccagaaaacagc‐3′
SIRT1	5′‐tgtttcctgtgggatacctga‐3′	5′‐tgaagaatggtcttgggtcttt‐3′
FOXO1	5′‐catgcacagcaaacttcttcagt‐3′	5′‐agatgtgtgaggcatggtgttc‐3′
GAPDH	5′‐agacagccgcatcttcttgt‐3′	5′‐cttgccgtgggtagagtcat‐3′

### 2.9 Oxidative stress measurement

Myocardial tissue reactive oxygen species (ROS)  was detected by 10 μmol/L dihydroethidium (DHE, Sigma), protecting from light, after incubating in a 37°C incubator for 30 min, the ROS level was detected by a fluorescence microscope with red fluorescence and quantified using the ImageJ software.

NRVMs were processed as group divided. Superoxide dismutase (SOD) and malondialdehyde (MDA) detection kits (Nanjing Jiancheng Bioengineer Institute) were following the manufacturer's instructions used for the measurement of ROS level. A full‐wavelength microarray (Thermo Fisher Scientific) with at 450 nm and 532 nm wavelength detected the absorbance and correction by protein concentration which analysed by the BCA standard curve.

### 2.10 TUNEL staining and immunohistochemistryanalysis

After 24 h of reperfusion, pre‐chilled PBS buffer was used to wash away blood from the myocardium, then 4% paraformaldehyde fixation (4°C, 24 h), then dehydrated in graded ethanol, cleared with xylene and embedded in paraffin. The samples taken 2 mm below the ligation line were then serially sectioned at a thickness of 4 μm and performed using the DeadEnd™ Fluorometric TUNEL System (Promega, G3250) following the manufacturer's protocols. DAPI solution (Beyotime) was used to mark the nucleus. A Zeiss 800 confocal microscope (Zeiss Microsystems) was applied to detect the fluorescence.

Immunohistochemical staining was according previously described with slightly modification.^30^ 4% paraformaldehyde fixed the myocardium and then embedded in paraffin. p‐AMPK (1:20), SIRT1 (1:50) and p‐FOXO1 (1:100) were used as primary antibodies. Positive areas were evaluated by diaminobenzidine (DAB) staining. Images were taken by a microscope (ZEISS Imager.M2).

### 2.11 Western blotting analysis

RIPA lysis buffer with protease inhibitor and phenylmethanesulfonylfluoride (PMSF) were used to lyse myocardium tissues and cell sample. The Pierce™ BCA protein assay kit (Thermo Fisher Scientific) was used to detect total protein concentration based on a standard curve. 5× loading buffer were added into samples and boiled at 100°C for 5 min. Equal protein concentration (50 μg/tissue sample and 30 μg/cell sample) were separated, respectively, with 8%–10% SDS‐PAGE, then transferred to nitrocellulose membrane (NC) membranes (0.22 μm, BOSTER). NC membranes were blocked in tris‐buffered saline + Tween 20 (TBST) with 5% skimmed milk for 1 h at room temperature and then incubated with primary antibodies. p‐AMPK (1:1000), AMPK (1:1000), SIRT1(1:2000), p‐FOXO1(1:1000), FOXO1(1:1000), β‐actin (1:3000) and β‐Tublin(1:3000) at 4°C overnight. After wash the membrane 3 times, 10 min each time, membranes were incubated with secondary antibodies 1 h and signals were detected by a Pierce™ ECL Plus Substrate (Thermo Fisher Scientific).

### 2.12 Cell viability assessment

The viability of NRVMs and H9c2 cells were evaluated by the CCK‐8 Kit (Dojindo Laboratories). Cells were seeded in 96‐well plates, and 10 μl of detection solution was added to each well for detection and incubated for 2 h following the manufacturer's instructions. Cell viability was calculated by the absorbance of 450 nm with a full‐wavelength microarray (Thermo Fisher Scientific).

### 2.13 Statistical analysis

All data presentations are shown as mean ± standard deviation (SD) unless otherwise stated. Significance between two groups was performed by Student's two‐tailed *t*‐test with GraphPad Prism 6.01 (GraphPad Software). In other cases, significance for more than two groups was done using one‐way ANOVA in Prism 8. Differences were considered significant at *p* < 0.05.

## 3 RESULT

### 3.1 RSV mitigates the myocardial infarct size, improves cardiac function and decreases oxidative stress caused by MI/RI

**FIGURE 1:**
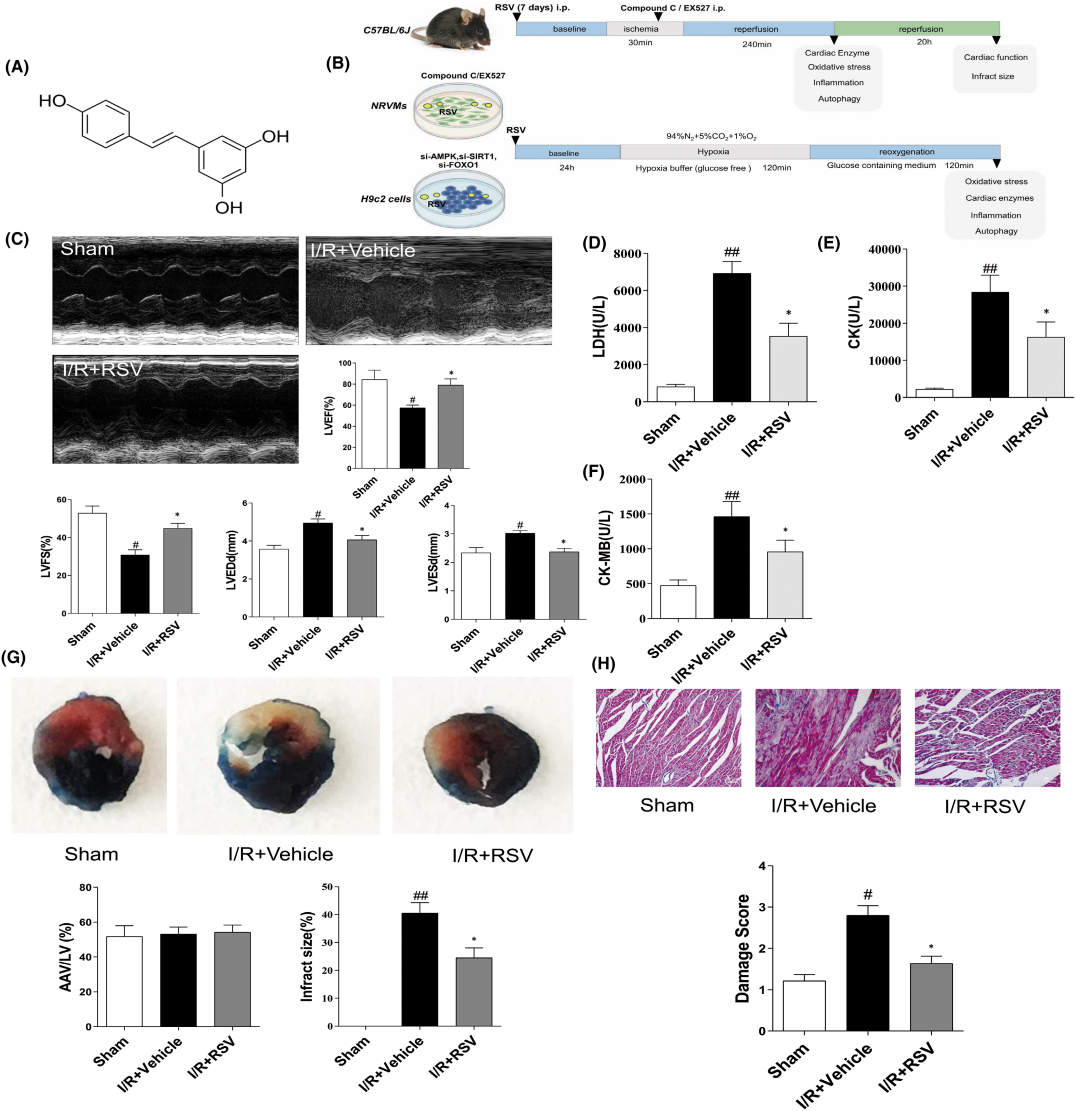
Changes in cardiac function and myocardium morphology in mice with or without resveratrol (RSV) treatment. (A) The chemical structure of RSV. (B) The experimental design of our work. To examinate the cardiac protective of RSV on MI/RI, the animals were divided into RSV treatment, I/R + Vehicle, and sham groups. To determine the mechanism of RSV, another set of animals was administered with either CpC or EX527 immediately at the end of a 30 min ischemia period. The H/R model was launched in NRVMs and H9c2 cells. CpC and EX527, si‐AMPK, si‐SIRT1 or si‐FOXO1 were applied to reveal the roles of AMPK/SIRT1‐FOXO1 axis on the protective effect of RSV. (C) Representative echocardiograms in M‐mode records of left ventricular (LV) and data analysis of LV ejection fraction (LVEF), fractional shortening (LVFS), LV end‐diastole diameter (LVEDd), and LV end‐systolic diameter (LVESd) (*n* = 6). (D–F) Myocardial enzymes LDH (D), CK‐MB (E) and CK (F) levels in serum of mice (*n* = 6). (G) Representative TTC–Evans Blue stained sections of hearts and quantitative analysis of the LV infarct size. Infarct size (%) is expressed as the percentage of infarct area relative to the total left ventricular area. The nonischemic section shown in blue area, red represent risk area, and the infarct region is stained white (*n* = 6). (H) Representative H&E staining of the left ventricular area. Scale bar = 50 μm (*n* = 5). Each group were shown as the mean ± SD; ^#^ *p* < 0.05, ^##^ *p* < 0.01 vs. Sham group; *p < 0.05, **p < 0.01 vs. I/R + Vehicle group

Murine MI/RI was eatablish to investigated protective effects of RSV (Figure 1A). RSV was pretreated to C57BL/6 mice before MI/RI, followed by assessment of indicators at various time phase. Infarct size was evaluated by Evens‐bule and TTC staining at 24 h after reperfusion. The results shown infarct size increased to 33% after MI/RI and was decreased to 20% after RSV pretreatment (Figure 1G).

To detect changes in cardiac function of MI/RI mice, echocardiography was performed. LVEF and LVFS were measured in a M‐mode to evaluate cardiac contractile function. We found MI/RI insult apparently reduced LVEF and LVFS compared with sham group, while RSV pretreatment tremendous increased LVEF and LVFS compared with I/R + Vehicle group (Figure 1C). Above data indicate that MI/RI led to cardiac dysfunction, and RSV pretreatment improve cardiac contractile function. However, cardiac function in I/R + RSV + CpC and I/R + RSV + EX‐527 group were reverse the cardioprotective of RSV, implying RSV via AMPK and SIRT1 pathway against I/R injury (Figure 3A). Our study suggests that RSV pretreatment via AMPK and SIRT1 pathway alleviate MI/RI‐induced reduction of cardiac contractile function, thereby protecting hearts from MI/RI.

**FIGURE 2:**
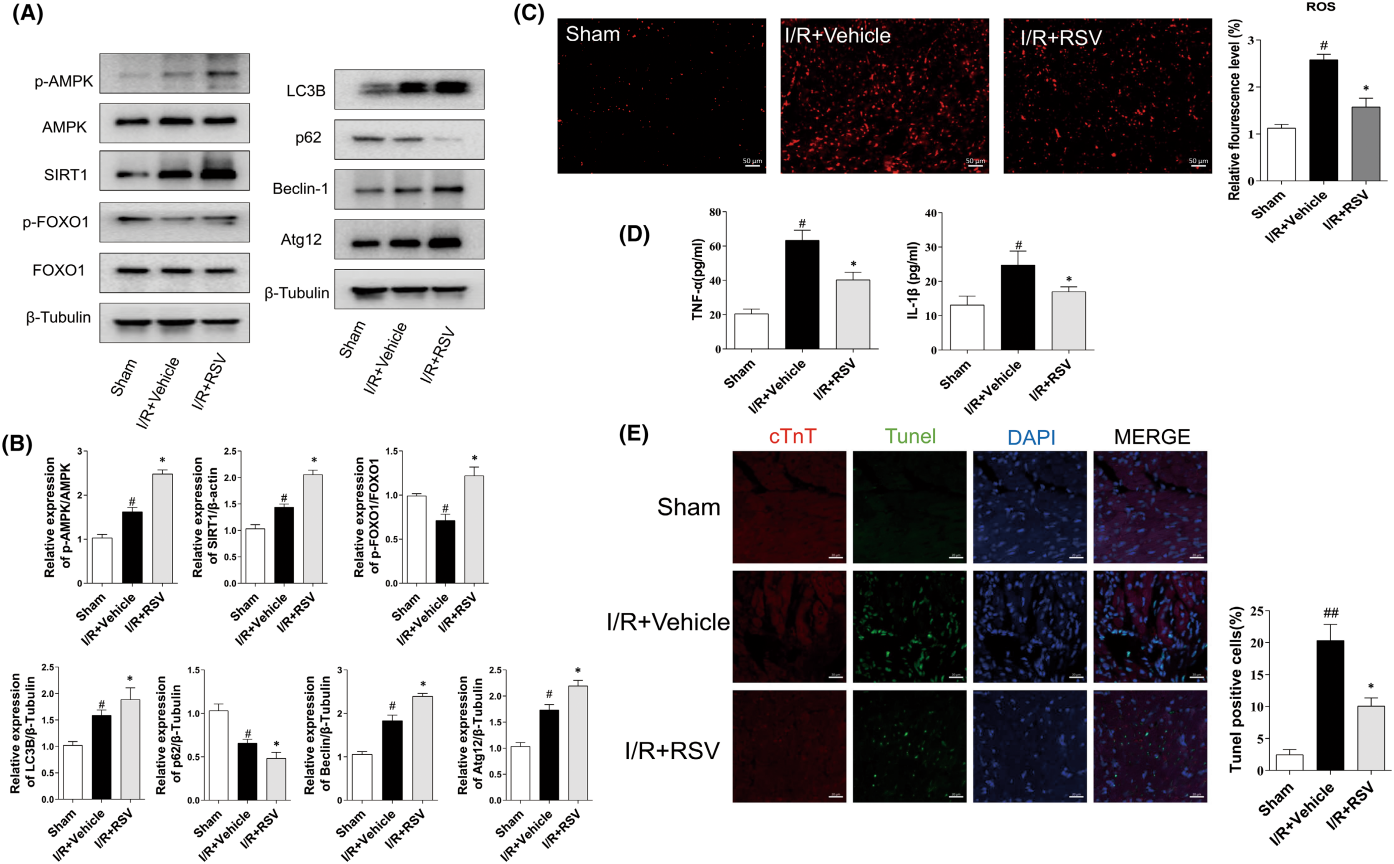
Resveratrol (RSV) alleviates cardiac damage caused by MI/RI. (A) The protein expression of p‐AMPK, AMPK, SIRT1, p‐FOXO1, FOXO1, LC3B, P62, Beclin‐1and Atg12 in myocardium tissue presented by representative Western blot bands (*n* = 5). (B) The protein expression p‐AMPK, AMPK, SIRT1, p‐FOXO1, FOXO1, LC3B, P62, Beclin‐1and Atg12 in myocardium tissue presented by statistical histograms of representative Western blots. (C) The representative DHE staining of the left ventricular of the mice, scale bar = 100 μm (*n* = 6). (D) Quantitative analyses of DHE staining level (*n* = 5). (E) Immunofluorescence TUNEL staining after RSV injection (*n* = 5); scale bar = 20 μm. Each group were shown as the mean ± SD; ^#^ *p* < 0.05, ^##^ *p* < 0.01 vs. Sham group; *p < 0.05, **p < 0.01 vs. I/R + Vehicle group

For further observe oxidative stress, myocardium tissue staining of DHE was performed in LV. As shown in Figure 2C, DHE intensity significantly increased after MI/RI insult and was also decreased after RSV treatment. However, compare with I/R + RSV group, CpC and EX‐527 significantly aggravate oxidative stress (Figure 4A). Together, our results further revealed that AMPK and SIRT1 were associated with RSV treatment on MI/RI‐induced myocardial infarction, cardiac dysfunction and oxidative stress.

### 3.2 RSV effect on the pathological changes and the levels of biochemical indicators in MI/RI mice

To evaluate the treatment of RSV on MI/RI mice, histochemical staining was adopted to analysis the pathological and histological changes of myocardium tissues. HE staining result reflect the myocardium of the sham group was intact and the myocardial fibres were neatly arranged with well‐defined boundaries and bundled distribution. The morphology of nucleus has a normal shape and is evenly distributed in the cardiac tissue (Figure 1H). While in I/R + Vehicle group, it was observed that the myocardial fibres out of order, the horizontal stripes are gone, the cardiomyocytes were immersed with neutrophils, and the volume of cells were expanded, ruptured and died. The shape of the cell nucleus changes, and the distribution becomes uneven. RSV alleviate the above symptoms (Figure 1H), but CpC or EX‐527 reverse the treatment effect of RSV (Figure 4B).

**FIGURE 3:**
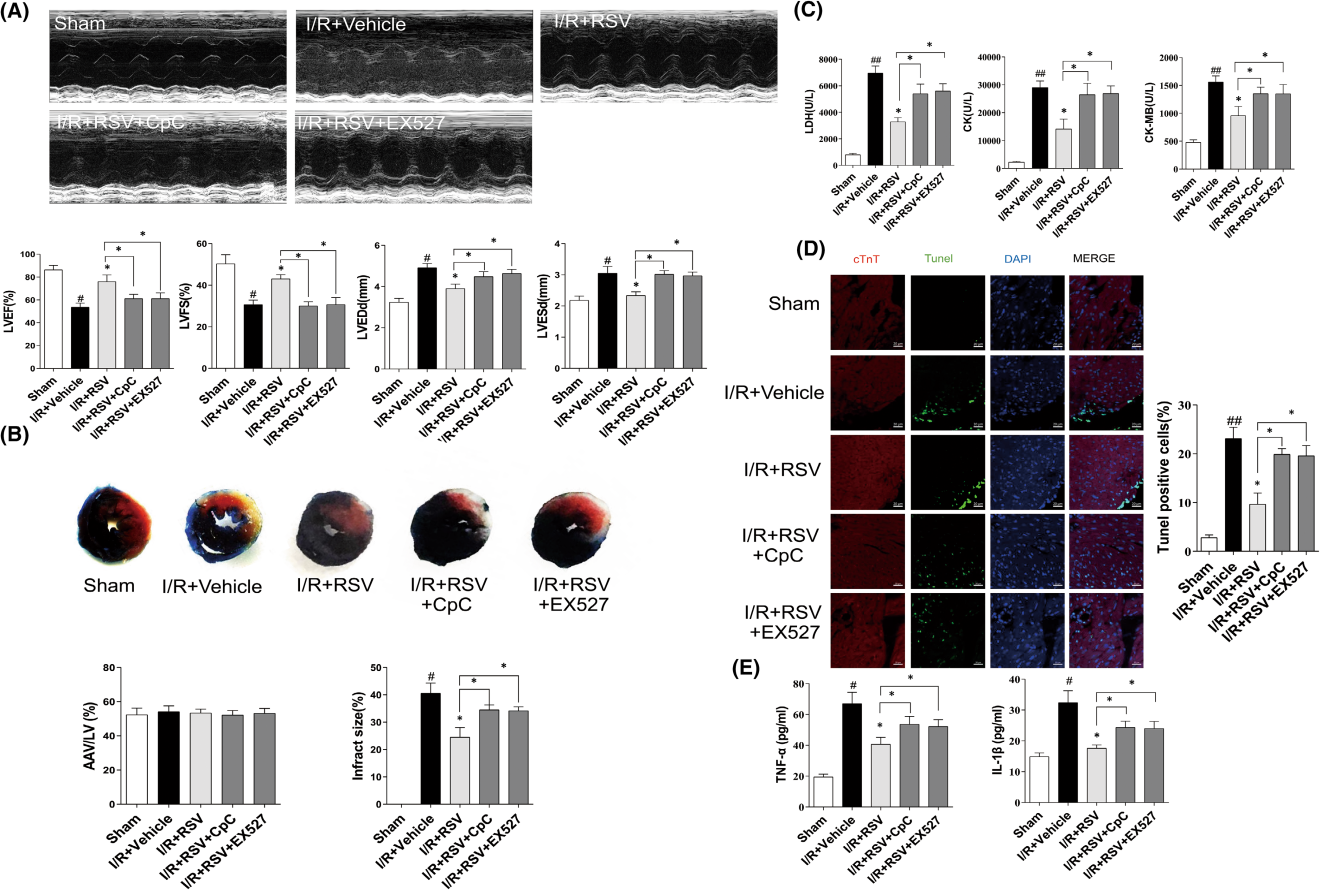
CpC or EX527 counteracted the protective effects of resveratrol (RSV) in vivo. (A) Quantitative assessment of left ventricular (LV) ejection fraction (LVEF), fractional shortening (LVFS), left ventricular end‐diastole diameter (LVEDd), and left ventricular end‐systolic diameter (LVESd) (*n* = 6). (B) Representative TTC–Evans Blue stained sections of hearts and quantitative analysis of the LV infarct size. (*n* = 6). (C) Quantification of myocardium tissue (LDH, CK, CK‐MB) on plasma of MI/RI mice treated with CpC + RSV or EX527 + RSV (*n* = 6). (D) Immunofluorescence TUNEL staining after CpC + RSV or EX527 + RSV treatment (*n* = 6). (E) TNF‐α and IL‐1β in myocardium tissue level were detected by ELISA assays (*n* = 5). Each group were shown as the mean ± SD; ^#^ *p* < 0.05, ^##^ *p* < 0.01 vs. Sham group; *p < 0.05, **p < 0.01 vs. I/R + Vehicle group

Furthermore, contrasted with the Sham group, serum contents of LDH (Figure 1D), CK (Figure 1E) and CK‐MB (Figure 1F) levels apparently increased in I/R + Vehicle group, suggesting that cardiomyocytes were injured during MI/RI insult. RSV pretreatment suppress the activities of LDH and CK‐MB and CK. However, the effect of RSV on biochemic indicate of LDH, CK, CK‐MB levels were reversed in RSV + CpC or EX‐527 group(Figure 3C). Together, these results suggest that RSV effectively lessen histopathological necrotic areas and resist myocardium damage caused by MI/RI.

### 3.3 RSV activating the AMPK, SIRT1 signalling pathway promoting expression of autophagy and decrease apoptosis level

Apoptosis of cardiomyocytes is a direct terminal indicator and plays a crucial role of MI/RI. In the present study, we used TUNEL staining to detect apoptotic cells. As shown in Figure 2E. Compared with the Sham group, the green fluorescence intensity of myocardial tissue was apparently increased in the IR + Vehicle group. After RSV pretreatment, green fluorescence intensity was significantly reduced. Together, these findings indicate that RSV reduces cardiomyocyte apoptosis. However, in the I/R + RSV + CpC and I/R + RSV + EX‐527 group, the fluorescence was increased compared with the I/R + RSV group (Figure 3D).

Next, compared with Sham group, protein expression of p‐AMPK/AMPK, SIRT1, LC3B Beclin‐1 and Atg12 significantly upregulated, while p‐FOXO1/FOXO1, p62 were downregulated in the I/R + Vehicle group. In the RSV treatment group, p‐AMPK/AMPK, SIRT1, p‐FOXO1/FOXO1, LC3B, Beclin‐1and Atg12 were further increased, while and p62 were decreased (Figure 2A).

**FIGURE 4:**
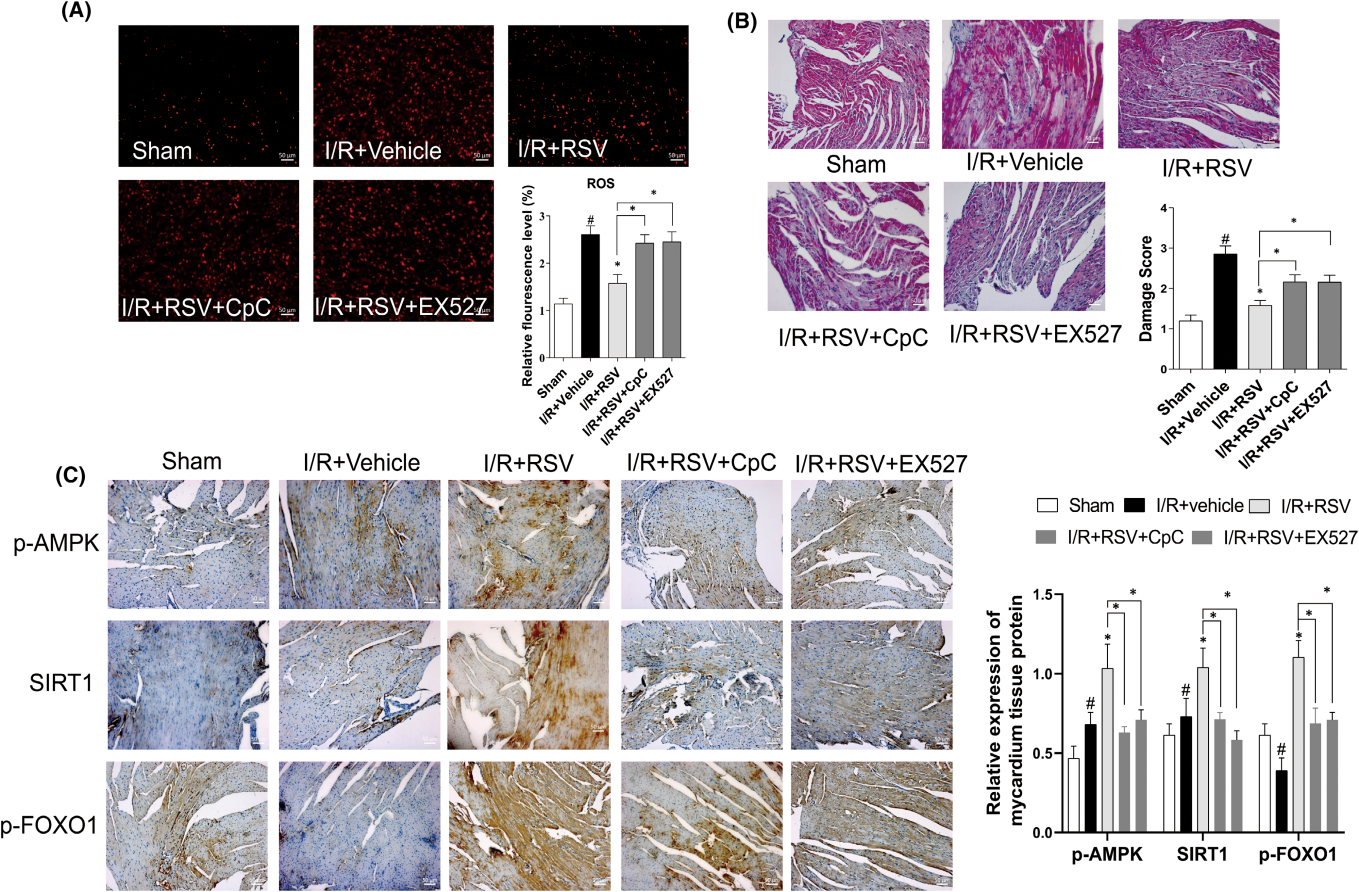
Resveratrol (RSV) cardioprotective effects is dependent on AMPK, SIRT1. (A) ROS level in myocardium detected by DHE staining. (B) Representative images of the mice myocardium prepared for histological evaluation under a light microscope with H&E staining. (C) Immunohistochemistry detected the expression of SIRT1, p‐AMPK, p‐FOXO1in the myocardium tissue. Each group were shown as the mean ± SD; ^#^ *p* < 0.05, ^##^ *p* < 0.01 vs. Sham group; *p < 0.05, **p < 0.01 vs. I/R + Vehicle group, *n* = 6

We then performed immunohistochemical staining to evaluate the expression of p‐AMPK, SIRT1 and p‐FOXO1 in myocardial tissue. The result of immunohistochemical staining shown cardiac myocardial sections in all groups consistent with Western blot. In the sham group, p‐AMPK was mainly expressed in the nucleus. SIRT1 was expressed both in the cytoplasm and nucleus. p‐FOXO1 expressed mainly in the cytoplasm. The analysis suggested that the expressions of AMPK, SIRT1 in I/R + Vehicle group were upregulated than the Sham, while p‐FOXO1decreased. RSV further increased the expressions of AMPK, SIRT1 and p‐FOXO1 compared with the I/R + Vehicle group (Figure 4C). Together, these data suggest that RSV activating p‐AMPK and promoting autophagy, this reaction could be reversed by CpC and EX‐527 treatment.

### 3.4 RSV suppress the expression of pro‐inflammatory factors by regulating AMPK, SIRT1 activation

In our study, we observed the effect of RSV on MI/RI‐induced pro‐inflammatory cytokine release. Inflammatory factor of TNF‐α and IL‐1β in cardiac tissue homogenates were detected by ELISA kits. As Figure 2D shown, compared with the sham group, the concentration of TNF‐α and IL‐1β were apparently increased due to MI/RI insult. Compared with the I/R + Vehicle group, the concentration of TNF‐α and IL‐1β were decreased in the I/R + RSV group. However, RSV co‐administrated with CpC or EX‐527, the levels of TNF‐α and IL‐1β in the I/R + RSV + CpC or I/R + RSV + EX‐527 group were higher than those in the I/R + RSV group (Figure 3E). Together, these data suggest that RSV via AMPK, SIRT1 inhibited pro‐inflammatory factors of TNF‐α and IL‐1β.

### 3.5 RSV alleviate the cell damage caused by H/R in NRVMs

**FIGURE 5:**
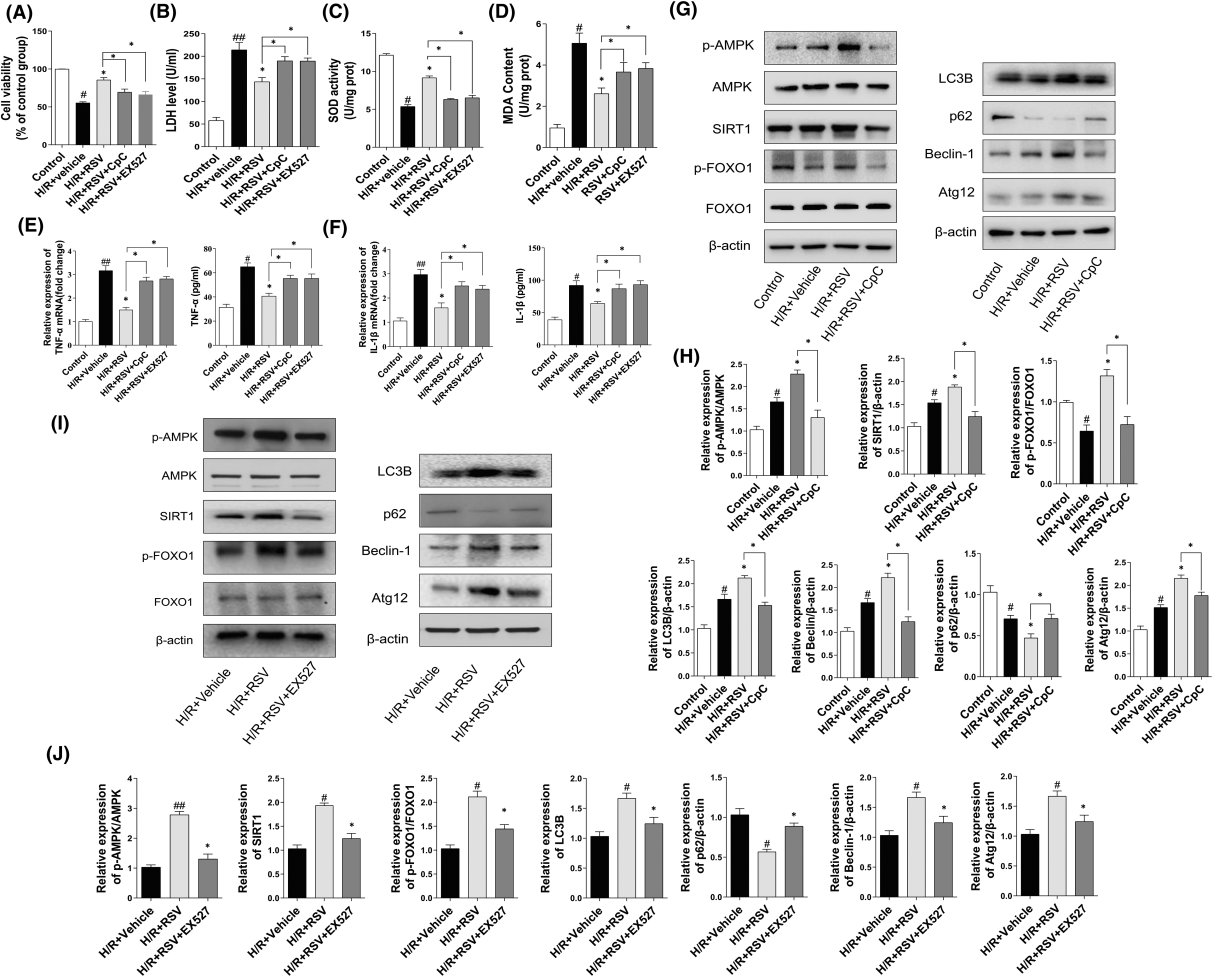
Resveratrol (RSV) ameliorates the cell injuries caused by H/R in NRVMs. (A) CCK‐8 assay detect cell viability of NRVMs (*n* = 3). (B) Cell supernatants of LDH leakage (*n* = 3). (C, D) SOD activity and MDA contents of NRVMs assayed with a commercial kit (*n* = 3). (E, F) TNF‐α and IL‐1β level in cell supernatants were determined by ELISA assays and TNF‐α mRNA and IL‐1β mRNA level. (G) Western blotting detected the protein expression of p‐AMPK, AMPK, SIRT1, p‐FOXO1, FOXO1, LC3B, P62, Beclin‐1 and Atg 12 after treatment with CpC + RSV (*n* = 3). (H) The protein expression of p‐AMPK, AMPK, SIRT1, p‐FOXO1, FOXO1, LC3B, P62, Beclin‐1 and Atg 12 presented by statistical histograms of representative Western blots. (I) Western blotting detected the protein expression of p‐AMPK, AMPK, SIRT1, p‐FOXO1, FOXO1, LC3B, P62, Beclin‐1 and Atg 12 after treatment with EX527 + RSV (*n* = 3). (J) The protein expression of p‐AMPK, AMPK, SIRT1, p‐FOXO1, FOXO1, LC3B, P62, Beclin‐1 and Atg 12 presented by statistical histograms of representative Western blots. Each group were shown as the mean ± SD; ^#^ *p* < 0.05, ^##^ *p* < 0.01 vs. Sham group; *p < 0.05, **p < 0.01 vs. I/R + Vehicle group

To observe the cardioprotective of RSV, an in vitro H/R model of NRVMs was established. Notably, pretreatment with RSV significantly increased the cell viability (Figure 5A). LDH release is a key indicator of cell membrane integrity. Our results indicated that H/R led to excessive LDH leakage in the supernatant, while RSV could significantly suppress the increase of LDH and ameliorate cell damage (Figure 5B). The activity of SOD and MDA content, two direct indexes of oxidative stress. SOD activity decreased in H/R‐exposed cells and was increased after RSV treatment (Figure 5C), while MDA content increased in H/R‐induced cell damage was reversed RSV (Figure 5D). Taken together, these data revealed that RSV significantly alleviates cell oxidative stress and cell injury caused by H/R in NRVMs.

### 3.6 RSV ameliorate I/R injury by activating AMPK, SIRT1 signalling pathway in NRVMs

In the present study, RSV was observed to activate AMPK and SIRT1 signalling, restore cardiac function and reduce myocardial infarct size after MI/RI. To evaluate the effect of resveratrol, the RSV pretreatment NRVMs into normal culture, H/R + Vehicle intervention, H/R + RSV intervention group (Control, H/R + Vehicle, H/R + RSV). Consistent with our animal data, compared with control group, H/R + Vehicle group the expression of p‐AMPK/AMPK, SIRT1 were obviously elevated, while p‐FOXO1/FOXO1 was decreased. Compared with H/R + Vehicle group, H/R + RSV further promoted the increase of p‐AMPK/AMPK, SIRT1, p‐FOXO1/FOXO1 (Figure 5G–J). However, RSV co‐administrated with CpC or EX‐527, the effect of RSV on p‐AMPK, SIRT1 and p‐FOXO1 were reversed in the group of I/R + RSV + CpC or I/R + RSV + EX‐527 (Figure 5G,I). These results indicated that RSV protects cardiomyocytes from H/R damage via AMPK/SIRT1 pathway.

### 3.7 Cardioprotective effects of RSV is dependent on AMPK or SIRT1 and blocking inflammation factors

To explore the mechanism by which RSV alleviate MI/RI, we detect the autophagy level, manifested by the expression of Beclin‐1, LC3B, p62 and Atg12. As shown in Figure 5 F, H, Beclin‐1, LC‐3B and Atg12 were apparently increased in the H/R + Vehicle group compared with the control group, while p‐62 was decreased. Moreover, the treatment of RSV further enhanced Beclin‐1, LC‐3B and Atg12 expressions and decreased the expression of p62. Meanwhile, treatment with AMPK inhibitor CpC or SIRT1 inhibitor EX‐527 counteracted the effect induced by RSV (Figure 5G–J).

Furthermore, the effect of RSV on inflammation factor was further observed in NRVMs. Consistent with the animal results, TNF‐α and IL‐1β in all groups from NRVMs showed a similar trend. The level of TNF‐α and IL‐1β in the cell supernatant was obviously increased after H/R. However, the treatment of RSV decreased the level of TNF‐α and IL‐1β (Figure 5E,F). In addition, CpC or EX‐527 application blocked the effect of RSV. TNF‐α, IL‐1β mRNA levels shown similar trends (Figure 5E,F). Together, above data indicated that RSV promoted autophagy level, demonstrated by upregulating the expression of Beclin‐1, LC‐3B and Atg12 but decreased the expression of p62 and suppressed the inflammation factor of TNF‐α and IL‐1β.

### 3.8 Related factors expression of knocking down AMPK, SIRT1 and FOXO1 in H/R cultured H9c2 cells

**FIGURE 6:**
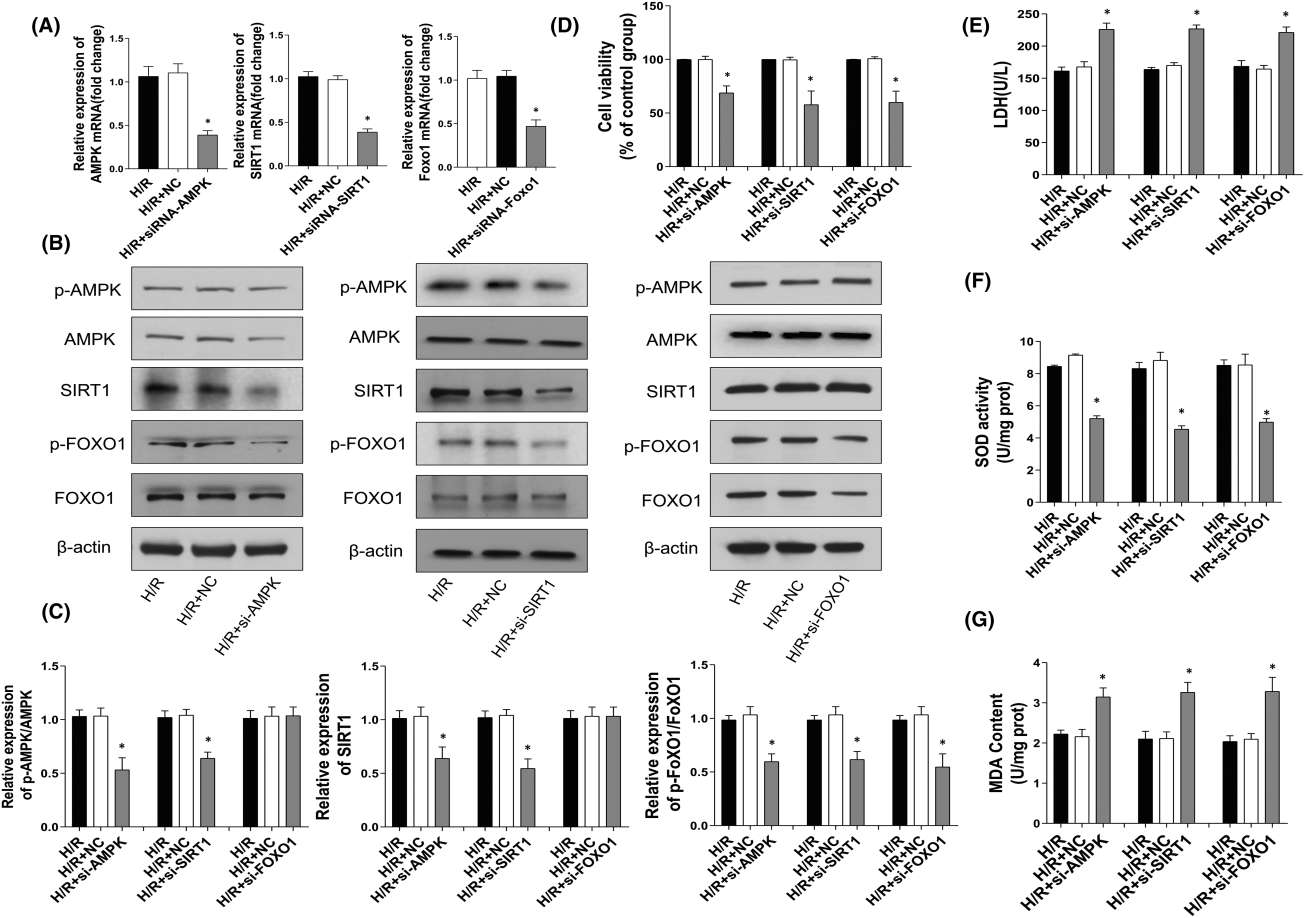
Related factors expression of knocking down AMPK, SIRT1 and FOXO1 in H/R cultured H9c2 cells. After application of AMPK, SIRT1 and FOXO1siRNA or negative control siRNA (NC siRNA) for 48 h, H9c2 cells were exposed to hypoxia for another 2 h, and subsequently reoxygenation for 2 h. (A) mRNA levels of AMPK, SIRT1 and FOXO1 in H9c2s were evaluated by RT‐PCR at 24 h post‐transfection. (B) Western blots detect protein expression of p‐AMPK, AMPK, SIRT1, p‐FOXO1, FOXO1. (C) The protein expression levels of p‐AMPK, AMPK, SIRT1, p‐FOXO1, FOXO1 presented by statistical histograms of representative Western blots. (D) CCK‐8 assay detect cell viability of H9c2 cells. (E) Cell supernatants of LDH leakage (*n* = 3). (F, G) SOD activity and MDA contents of H9c2 cells assayed with a commercial kit (*n* = 3). Each group are presented as the mean ± SD; ^#^ *p* < 0.05, ^##^ *p* < 0.01 vs. Sham group; *p < 0.05, **p < 0.01 vs. I/R + Vehicle group

In order to illustrate the regulating role of AMPK, SIRT1 and FOXO1, H9c2 cells were cultured into various groups including H/R, H/R + siRNA normal control, H/R + AMPK, SIRT1 or Foxo1 siRNA transfected groups (H/R, H/R + si‐NC, H/R+ si‐AMPK, H/R + si‐SIRT1 and H/R + si‐Foxo1). Real‐time PCR and Western blot results shown that the expression of AMPK or SIRT1 was significantly inhibited after transfection of AMPK or SIRT1 siRNA and p‐FOXO1/FOXO1 were lower than those in the H/R group (Figure 6A–C). Compared with the H/R + NC group, the protein expression of p‐FOXO1/FOXO1 in the H/R + si‐FOXO1 group was obviously decreased, but there was no significant difference in p‐AMPK/AMPK and SIRT1 levels. In addition, CCK‐8 results (Figure 6D) indicated that cell viability was decreased in H/R + si‐AMPK, H/R + si‐SIRT1 and H/R + si‐FOXO1 groups and LDH results (Figure 6E) further verify that. Compared with H/R + NC group, LDH released increased in H/R + si‐AMPK, H/R + si‐SIRT1 and H/R + si‐FOXO1 group. Moreover, SOD activity decreased in H/R + si‐AMPK, H/R + si‐SIRT1 and H/R + si‐FOXO1 group (Figure 6F), while MDA content increased (Figure 6G). Furthermore, there were no significant changes of mRNA and protein expression between H/R and H/R + si‐NC groups, excluding the effect of transfection on H9c2 cells.

## 4 DISCUSSION

In our study, we demonstrate that RSV reduces MI/RI in a mouse model as manifested by significantly reducing infarct size, improving cardiac function and reducing apoptosis. Meanwhile, we explored new signalling pathways and provided a new perspective on the prevention and treatment of MI/RI. Firstly, we found that resveratrol tremendous enhanced the expression of AMPK/SIRT1. Then we verified that RSV could reduce oxidative stress level and induce autophagy through AMPK/SIRT1‐FOXO1 signalling pathway in vivo and in vitro. Finally, we further examined the relationship between AMPK, SIRT1 and FOXO1, we adopted pharmacological methods and siRNA knockdown the expression of AMPK, SIRT1 and FOXO1. Our research put forward a new theoretical basis for studying the mechanism and potential targets of MI/RI.

Previous studies have shown that RSV possessed anti‐oxidative stress, inhibit inflammatory, and reduce cellular apoptosis properties that plays a therapeutic role in various of diseases.^31^, ^32^, ^33^ Our DHE staining results shown that MI/RI led to the excessive generation of ROS, while RSV significantly suppressed the production of ROS myocardium tissue, and the levels of oxidant stress enzymes, such as SOD activity was upregulated after RSV treatment in NRVMs and MDA contents were decreased in RSV treatment group in comparison with H/R+ Vehicle group. These results demonstrated that RSV exert myocardial protection effects partly through alleviate oxidative stress.

Autophagy is usually defined as a survival program that occurs under stress conditions, and the ability to remove damaged proteins or organelles to maintain the normal function of mitochondria is related to cell protection and internal environment stability, but excessive autophagy affects the body is harmful.^34^, ^35^ Previous studies suggest that autophagy has a protective effect on myocardial injury after myocardial infarction, and further activation of autophagy after reperfusion may accelerate myocardial injury; therefore, appropriate up‐regulation of autophagy can play a cardioprotective effect during myocardial ischemia–reperfusion injury.^36^, ^37^ Xianfeng Qu et al.^38^ shown that RSV can via regulating autophagy level to restore cardiac dysfunction in diabetic mice. In addition, research showed that the level of autophagy induced by reperfusion injury may be directly related to the degree of ischemia.^36^ If it is gentle, the induction of enhanced autophagy during the reperfusion phase is beneficial. Conversely, hyperactivation of autophagy leads to the clearance of essential proteins or organelles in cells, resulting in cell dysfunction and apoptosis. Furthermore, autophagy is associated with inducing factors, such as hypoxia and lack of glucose, which may lead to opposite effects.

Beclin‐1 as the mammalian homologue of yeast Atg6, playing an vital role in initiating autophagy and Beclin‐1 up‐regulation during MI/RI process promoted autophagosome formation.^39^ Moreover, Atg genes regulate Atg12‐Atg5 and LC3B complexes to regulate autophagosome formation.^40^ In addition, LC3B was crucial in the whole process of autophagy, and their expression levels reflect the process of autophagy.^41^ Moreover, late autophagy degradation product of p62, an autophagy receptor protein, is negatively correlated with autophagy activity.^42^ In our study, RSV activated Beclin‐1 and LC3B expression, and the autophagic flux suppression by CpC and EX527 counteracted the effect of RSV‐induced autophagy activation. Our results were consistent with previous studies suggest that RSV induces autophagy and can be demonstrated by the ability to activate the autophagy markers LC3B and Beclin‐1 against spinal cord injury.^43^ Meanwhile, we also found RSV could decrease I/R‐induced pro‐inflamation factor relaese through AMPK, SIRT1‐dependent autophagy activity. Previous research has suggested that increased levels of TNF‐α and IL‐1β could result in severe organ injury by enhancing the inflammatory response.^44^ Our study suggested that RSV decreased TNF‐α and IL‐1β level caused by MI/RI. In summary, these results provide new proof that RSV induces autophagy and alleviate inflammation in MI/RI process.

Moreover, we found RSV exert cardioprotective effect via AMPK/SIRT1‐FOXO1 signal pathway in vivo. To further observe the role of RSV and AMPK/SIRT1‐FOXO1pathway during MI/RI, NRVMs and H9c2 cells (foetal rat cardiomyocyte‐derived cell line) were used in vitro experiments. Promoting AMPK activation and further activate SIRT1 by upregulating intracellular NAD+ level. AMPK and SIRT1 feel changes in the cellular environment through AMP/ATP energy state and NAD+/NADH redox state, and affect cellular function through FOXO1 phosphorylation and deacetylation, respectively. Moreover, SIRT1 participated in multiple important biological functions, such as energy metabolism, oxidative stress, autophagy and apoptosis.^45^, ^46^

Transcription factor FOXO family, which plays a crucial role in cell proliferation, DNA repair and cell cycle block.^47^ Among them, FOXO1 is a key regulator that related with oxidative stress, inhibited autophagy and promoted apoptosis in animal models of MI/RI.^48^ RSV increased phosphorylation of FOXO1 through phosphorylation of AMPK and SIRT1, thereby reducing ROS and apoptosis level to alleviate myocardial ischemia–reperfusion injury. In addition, AMPK and SIRT1 regulate autophagy as nutritional sensory signals in MI/RI. Under nutrient deprivation or ischemia and hypoxia condition can promote autophagy level by activating AMPK and SIRT1.^49^ In addition, Xiaomu Liu et al.^50^ reported that RSV could activate the AMPK‐SIRT1 signalling pathway to induces apoptosis and inhibits adipogenesis in bovine intramuscular adipocytes. In addition, activation of SIRT1 can negatively regulate FOXO1‐dependent apoptosis related gene transcription t and cell cycle arrest to protect endothelial cells.^51^ In the present study, EX‐527 counteracted the cardiac protective of RSV on SIRT1 expression, AMPK activation and autophagy activity. CpC remarkably decreased SIRT1 expression suggested AMPK and SIRT1 were interacting with each other. And EX‐527, CpC significantly upregulated the expression of p62 and downregulated level of LC3B compared with the I/R + RSV groups. Our data indicate that AMPK/SIRT1‐FOXO1 signalling pathway related with ROS and autophagy level in MI/RI process.

To further explore the pathological mechanism between AMPK, SIRT1 and FOXO1, we adopted gene silence with siRNA method to knockdown the expression of AMPK, SIRT1 and FOXO1 in H9c2 cells. Our data suggest that p‐AMPK, SIRT1 were increased and p‐FOXO1/FOXO1 was decreased in NRVMs and MI/RI mice. Knock down the expression of AMPK and SIRT1 can decreased the protein expression to each other and down‐regulate the expression of p‐FOXO1, while knock down FOXO1 expression cannot affect the level of p‐AMPK and SIRT1. Our study suggest that AMPK can regulate downstream molecules SIRT1 and FOXO1 expression, and SIRT1 can also regulate the expression of AMPK and FOXO1. However, this study did not investigate the specific regulatory mechanism of SIRT1 on AMPK. The study of Ruderman et al. have reported that cardiac SIRT1 has been shown to deacetylate LKB1 to mediate AMPK activation, which regulates SIRT1 activity during ischemia by regulating NAD+ levels.^52^

## 5 CONCLUSION

**FIGURE 7:**
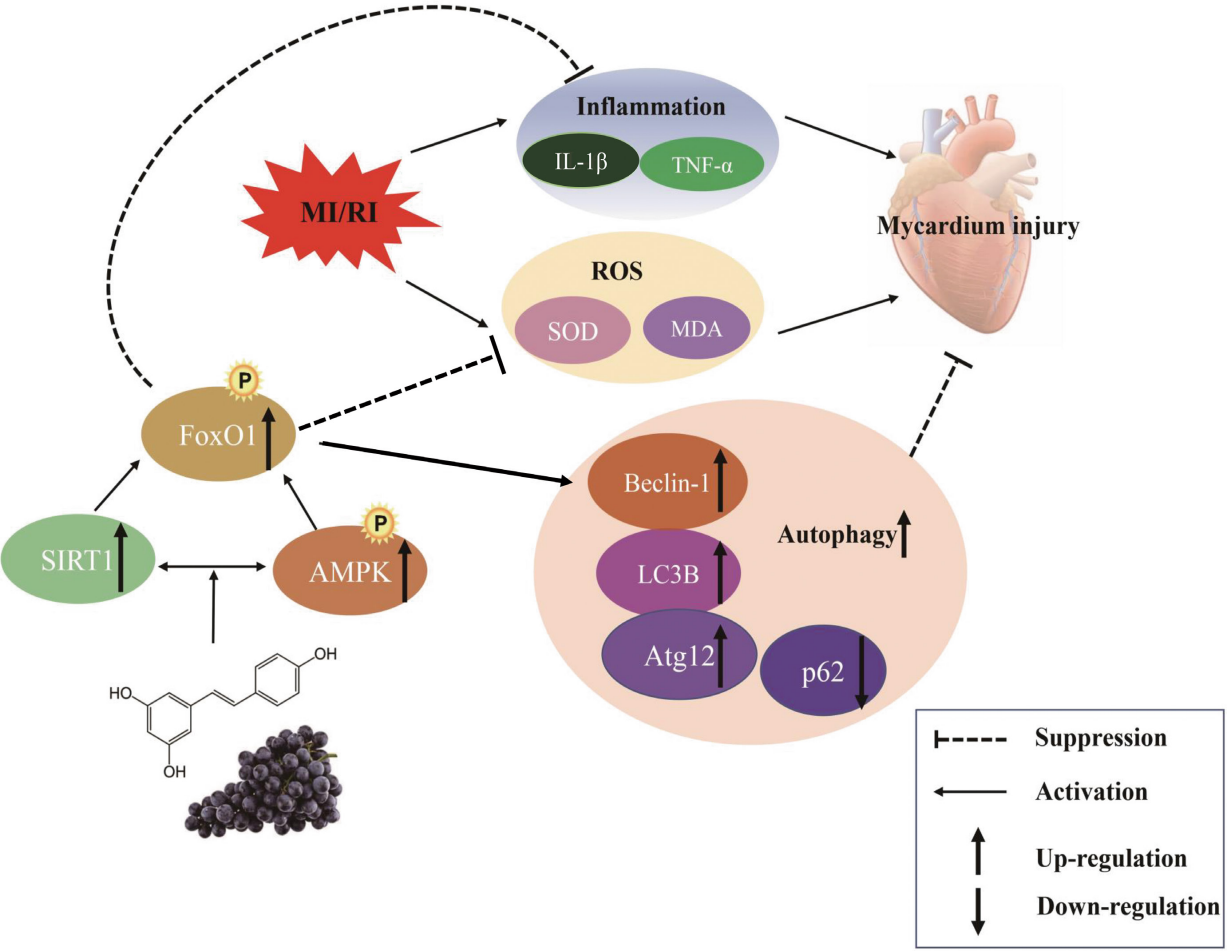
Underlying molecular mechanisms of the protective effects of resveratrol (RSV) against the myocardial ischemia/reperfusion injury of mice. Scheme summarizing the protective effects of RSV preconditioning attenuates MI/RI via activating of AMPK/SIRT1‐FOXO1 signalling and autophagy. MI/RI amplifies inflammation and ROS overproduction, Importantly, RSV upregulated the AMPK signalling pathway, contributing to inhibit MI/RI‐induced inflammation and oxidative stress and induced autophagy to mitigate MI/RI

We found that RSV can significantly ameliorate ROS level, inflammation and apoptosis after MI/RI in vivo and in vitro by regulating AMPK/SIRT1‐FOXO1 pathways. Meanwhile, we identified that RSV induce autophagy to mitigate MI/RI. The possible molecular mechanisms are shown in Figure 7. The results provide direct evidence for further study of the protective effect of RSV in myocardial tissue and development of efficient strategies to activate the SIRT1‐AMPK/FOXO1 pathway may eventually lead to improving the survival of MI/RI patients.

## AUTHOR CONTRIBUTIONS

**Haiyan Li:** Conceptualization (equal); project administration (equal); writing – original draft (lead). **Fuchun Zheng:** Data curation (equal); methodology (equal); resources (equal); validation (equal); writing – original draft (equal). **Yanmei Zhang:** Data curation (supporting); formal analysis (equal); investigation (equal); methodology (supporting). **Jiajia Sun:** Data curation (supporting); methodology (supporting); project administration (equal). **Fenfei Gao:** Methodology (supporting); resources (supporting); validation (supporting). **Ganggang Shi:** Conceptualization (equal); funding acquisition (equal); project administration (equal); supervision (equal).

## CONFLICT OF INTEREST

The authors confirm that there are no conflicts of interest.

## Data Availability

The data that support the findings of this study are available from the corresponding author upon reasonable request.
